# Diversity and Spatiotemporal Atlas of Ticks in the Beijing–Tianjin–Hebei Urban Agglomeration Based on the MaxEnt Model

**DOI:** 10.3390/vetsci13070651

**Published:** 2026-07-03

**Authors:** Lingling Chen, Wanying Gao, Yang Song, Zihao Huang, Jialing Long, Jiaqi Nie, Zengliang Wang, Shulei Jia

**Affiliations:** 1 School of Basic Medical Sciences, Tianjin Medical University, Tianjin 300070, China; chenlingling2580@126.com (L.C.); hzh2023@tmu.edu.cn (Z.H.); long-jialing@tmu.edu.cn (J.L.); zhengguiliang2020@126.com (J.N.); 2 Institute of EcoHealth, School of Public Health, Cheeloo College of Medicine, Shandong University, Jinan 250012, China; wangzengliang@mail.sdu.edu.cn; 3 Chinese Center for Disease Control and Prevention, Beijing 102206, China; songyang@chinacdc.cn

**Keywords:** ticks and tick-borne diseases, MaxEnt model, Beijing–Tianjin–Hebei (BTH) region, suitable habitat, environmental factors

## Abstract

Background: This study aims to delineate the present and projected suitable habitats for four dominant tick species in the Beijing–Tianjin–Hebei (BTH) region, providing a spatial basis for targeted tick-borne disease surveillance. Methods: We systematically reviewed the published literature and the Global Biodiversity Information Facility (GBIF) to compile tick occurrence records in the BTH region. A total of 167 geo-referenced occurrence records with verified coordinates were obtained for four dominant species: *Haemaphysalis longicornis*, *Haemaphysalis concinna*, *Dermacentor silvarum*, and *Ixodes persulcatus*. The MaxEnt model was applied with bioclimatic variables (WorldClim, 2.5 arc-min), elevation, slope, aspect, and NDVI. Model performance was evaluated using the area under the receiver operating characteristic curve (AUC) for within-species comparisons, complemented by the True Skill Statistic (TSS), Cohen’s kappa, and omission rate. Future projections (2021–2040, 2041–2060, 2061–2080, 2081–2100) were made under the SSP245 scenario using only climate variables, as the NDVI and topographic variables cannot be reliably forecast. Results: The four dominant tick species showed distinct distribution patterns: *Hae. longicornis* was widely distributed across the BTH region, whereas *Hae. concinna*, *D. silvarum*, and *I. persulcatus* were mainly found in the northern and northwestern mountainous areas. The primary environmental drivers were temperature, elevation, and the NDVI. MaxEnt models achieved good predictive performance (test AUC: 0.86–0.91; TSS: 0.72–0.88). Under future climate scenarios, suitable habitat centroids were projected to shift northwestward for *Hae. longicornis* (~57.6 km), *D. silvarum* (~71.1 km), and *I. persulcatus* (~50.0 km), and northeastward for *Hae. concinna* (~63.0 km) by 2081–2100. Conclusions: In this study, we identified current and future high-risk areas for four dominant tick species in the BTH region, providing a reproducible foundation for surveillance. Future projections should be interpreted with caution as they only account for climatic changes.

## Simple Summary

Ticks are small blood-sucking creatures that can spread diseases to humans and animals. As the climate changes, ticks may move into new areas, increasing the risk of disease. In this study, we wanted to find out where four common tick species live now and where they might move to in the future across the Beijing–Tianjin–Hebei region, a large urban area in northern China. We collected information from scientific papers and databases about where ticks have been found. Using computer modeling, we linked tick locations with environmental factors like temperature, greenness of vegetation, and elevation. Our results showed that one tick species is widespread across both flat and mountain areas, while the other three are mostly found in northern mountain regions. Temperature, elevation, and vegetation were the most important factors shaping tick habitats. Looking ahead to future climate scenarios, we found that suitable tick habitats are likely to shift northward or northwestward. These findings help public health officials identify high-risk areas and plan better tick surveillance and disease prevention strategies to protect local communities.

## 1. Introduction

Ticks are obligate hematophagous ectoparasites that infest domestic animals, wildlife, and humans, and they have been considered the second most important vectors of human diseases worldwide [1]. In China, 124 known tick species have been recorded, comprising 113 hard ticks (Ixodidae) and 11 soft ticks (Argasidae), revealing substantial heterogeneity in tick diversity across the country [1,2,3]. In addition to being major ectoparasites of animals, ticks serve as vectors and reservoirs for numerous zoonotic pathogens, including bacteria, viruses, and protozoa, with increasing diversity over the past 30 years [3,4,5], posing serious threats to both human and animal health [6,7,8]. The tick microbiome, comprising bacteria, viruses, protozoa, and their functional genes, critically modulates pathogen transmission, host immune responses, and antimicrobial resistance dissemination across the human–animal-environment interface. Recent studies have demonstrated that microbiome composition can influence pathogen acquisition, maintenance, and transmission efficiency, positioning it as a major determinant of tick-borne disease dynamics [9,10]. Elucidating the composition and bioactive potential of tick-associated microbiomes is therefore critical for advancing prevention strategies, including next-generation vaccines and targeted interventions, under the One Health paradigm. Global analyses of zoonotic pathogens show that antimicrobial resistance genes spread across hosts, environments, and regions, driven by climate and socioeconomic factors, underscoring the need for integrated surveillance of tick-borne diseases in anthropogenically modified landscapes [11]. In parallel, climate change has driven significant northward and altitudinal expansion of Ixodidae ticks in the Northern Hemisphere, with warming winters allowing survival at previously unsuitable higher latitudes and elevations [12,13]. These climate-driven shifts are particularly concerning in East Asia, where rapid environmental changes are reshaping vector distributions. Moreover, climate change can indirectly alter vegetation distribution, host abundance, human behavior, and land use, all of which influence tick density [8,14,15,16]. In addition, in peri-urban areas of China, rapid urbanization has created novel interfaces between humans, domestic animals, and wildlife, driving shifts in tick host communities and altering the landscape of tick-borne disease risk [17]. Understanding these interacting factors and integrating them with spatial distribution modeling of dominant tick species under current and future climates is therefore essential for effective prevention and control under the One Health paradigm.

Various models have been developed to predict species distributions, including the Maximum Entropy (MaxEnt) model, Classification and Regression Trees (CART), Generalized Linear Models (GLMs), Habitat models (HABITAT), Genetic Algorithm for Rule-set Prediction (GARP), and Bioclimatic models (BIOCLIM) [16,18]. Among these, MaxEnt has become one of the most widely used due to its solid theoretical foundation in ecological niche modeling. Based on the principle of maximum entropy, the model estimates a species’ potential habitat distribution from occurrence data and environmental variables, providing the least biased prediction that remains consistent with known information, even with limited data. The MaxEnt model is known for its high predictive accuracy, stability, and ease of interpretation, particularly when applied to species with sparse or discontinuous records [19,20], making it especially suitable for modeling the distribution of ticks and other vector species.

The BTH region, located in northern China, is the country’s largest urban agglomeration in this area and features complex topography and ecosystems, including plains, hills, and mountains. These diverse habitats, along with rich animal resources, provide ideal breeding grounds and refuges for tick populations. Several tick-borne diseases, including spotted fever group rickettsiosis and severe fever with thrombocytopenia syndrome (SFTS), have been documented in this region [21,22,23]. Recent advances in microbiome research have further highlighted the complexity of pathogen dynamics. In recent years, the BTH region has undergone rapid urbanization and population growth while also implementing strict environmental protection policies. This dual process has expanded the extent of human activity, increasing the likelihood of human exposure to ticks and tick-borne pathogens. Thus, applying the MaxEnt model in conjunction with ArcGIS spatial analysis to investigate the distribution and future trends of dominant tick species in the BTH region is of great significance for public health surveillance and disease prevention.

To systematically assess the risk landscape of tick-borne diseases in the Beijing–Tianjin–Hebei (BTH) region and to fill the gap in spatial distribution studies of the dominant tick species in this area, in this study, we provided a comprehensive overview of the spatial distribution of tick species across the BTH region. By integrating the MaxEnt model with ArcGIS spatial analysis, we predicted the potential suitable habitats and projected future distribution shifts in dominant tick species under both current and future climatic scenarios. These findings provide critical insights for regional tick-borne disease surveillance and risk assessment, establishing a scientific basis for the development of targeted prevention and control strategies.

## 2. Materials and Methods

### 2.1. Collection of Tick Geographical Distribution Data

The BTH urban agglomeration is located in the northern part of the North China Plain and encompasses 13 cities, including Beijing, Tianjin, and 11 cities in Hebei Province. Data on tick distribution were collected through literature retrieval and data extraction. We searched major scientific citation databases, including PubMed (https://pubmed.ncbi.nlm.nih.gov/, accessed on 25 July 2024), Web of Science, China National Knowledge Infrastructure (CNKI), and the Wanfang Database, using the keywords: “tick”, “Beijing”, “Hebei”, and “Tianjin”. The search covered all relevant publications available up to 25 July 2024. In addition, we retrieved occurrence records from the Global Biodiversity Information Facility (GBIF) (iNaturalist contributors, iNaturalist (2026). iNaturalist Research-grade Observations. iNaturalist.org. Occurrence dataset https://doi.org/10.15468/ab3s5x accessed via GBIF.org on 1 July 2026. https://gbif.org/occurrence/4908910223 accessed on 1 July 2024). We also consulted the authoritative monograph Fauna Sinica: Arachnida: Ixodida from the Fauna of China series. Relevant distributional data and collection locations were extracted using the same procedures. We manually reviewed the titles, abstracts, and full texts of the articles and selected those that reported tick distribution in the BTH region. A total of 40 eligible articles (33 in Chinese and 7 in English) were retained. From these, we extracted geographic location and coordinate information. If longitude and latitude were not explicitly provided, the Baidu Map Coordinate Picker (https://api.map.baidu.com/lbsapi/getpoint/index.html, accessed on 25 July 2024) was used to determine approximate coordinates based on the described locations. Among all the occurrence records, each record includes species name, collection locality (at county level or finer), geographic coordinates, and year of collection when available. Coordinates are accurate to within <1 km for village-level records and approximately 10–30 km for county-level centroids. The full set of occurrence data (including coordinates, collection year, and tick stage) is provided as Tables S1–S3.

To correct for sampling bias inherent in the compiled occurrence records, we applied spatial rarefaction using the SDMToolbox v2.5 plugin in ArcGIS with a minimum distance of 10 km to reduce the influence of spatially clustered records. Subsequently, a bias grid was generated via Gaussian kernel density estimation (bandwidth = 50 km) of all tick occurrence points, and this grid was incorporated into MaxEnt as a sampling bias file to weight background points according to sampling intensity, following the recommendations of recent methodological studies [24]. For each species, background points were randomly sampled from the accessible area (M), defined as the BTH administrative boundaries plus a 50 km buffer to account for potential dispersal beyond the study region; a total of 10,000 background points were generated per species using the bias grid as a sampling probability raster, such that background sampling intensity mirrored the spatial distribution of the occurrence records [19,20,24]. This two-step procedure mitigates overestimation of model performance and ensures that environmental response curves reflect ecological gradients rather than sampling artifacts.

### 2.2. Selection of Environmental Variables

Environmental variables used in this study included both bioclimatic and geographic factors. Nineteen bioclimatic variables (Bio1–Bio19) were obtained from the WorldClim database (http://www.worldclim.org) at a spatial resolution of 2.5 arc-min. Although WorldClim lacks direct humidity variables, it is the most widely used bioclimatic dataset, and its temperature variables are highly relevant for tick overwintering survival. To partially compensate for the lack of humidity data, we used the Normalized Difference Vegetation Index (NDVI) as a proxy, as it indirectly reflects moisture-related microclimatic and vegetation conditions that influence tick survival and questing.

To select predictors and address collinearity, we performed Pearson correlation analysis in R. For variable pairs with |r| > 0.8, the variable showing greater ecological relevance and higher preliminary MaxEnt contribution was retained. The final variable set had pairwise correlations < 0.7.

Geographic variables (elevation, slope, and aspect) were derived from the ASTER Global Digital Elevation Model (GDEM). Elevation was retained despite its correlation with temperature (r = −0.68 to −0.82) because local topography creates non-monotonic temperature relationships and independently modulates microclimates, host movement corridors, and vegetation composition, thus capturing fine-scale heterogeneity not represented by coarse bioclimatic layers [25,26].

NDVI data were obtained from the MODIS/Terra Vegetation Indices Monthly L3 Global 1km SIN Grid (MOD13A3) version 6.1 dataset (https://www.earthdata.nasa.gov/). The time range was January 2000 to December 2023, matching the period of tick records. Data were downloaded from the LPDAAC (Land Processes Distributed Active Archive Center) and processed using the MODIS Reprojection Tool (MRT). The pixel reliability layer was used to mask out clouds, ice, snow, and low-quality pixels (only pixels flagged as “good” or “marginal” were retained). The native 1 km resolution of MOD13A3 was resampled to 2.5 arc-min (~5 km) using bilinear interpolation in ArcGIS to match the climate data.

Vector data for provincial administrative boundaries in the BTH region were acquired from the National Geomatics Center of China (https://www.tianditu.gov.cn/) under the cartographic license number GS (2024) 0650, with the coordinate system set to GCS_WGS_1984.

### 2.3. Construction of the MaxEnt Model

For the dominant tick species identified, the MaxEnt (Maximum Entropy) model was applied to analyze the key environmental factors influencing their distribution in the BTH region. Model settings were as follows: feature classes were set to auto; the regularization multiplier was initially set to 1.0 and then tuned by five-fold cross-validation in the range 1.0–2.0; the final value of 1.5 was chosen because it gave the highest mean AUC while minimizing model complexity. The convergence threshold was 10^−4^ and the maximum number of iterations was 500. A total of 75% of the occurrence records were randomly selected as the training dataset, while the remaining 25% were used for testing. For each dominant species, ten bootstrap replicates were performed. The jackknife method was employed to evaluate the relative contribution of each environmental variable. Univariate response curves were constructed to determine the environmental suitability range for each species.

Model performance was assessed using several metrics:

AUC (Area Under the Curve) of the Receiver Operating Characteristic (ROC) curve-used only to compare different model settings for the same species, not for cross-species comparisons. Test AUC values were 0.88 for *Hae. longicornis*, 0.88 for *Hae. concinna*, 0.91 for *D. silvarum*, and 0.86 for *I. persulcatus*, respectively.

True Skill Statistic (TSS) = sensitivity + specificity − 1, calculated using the threshold that maximizes training sensitivity plus specificity.

Omission rate was defined as the proportion of training points predicted below the 10th percentile threshold, and Cohen’s kappa evaluated agreement between predicted and observed occurrences. Sensitivity exceeded 0.85, specificity exceeded 0.80, and kappa ranged from 0.72 to 0.88 for all species, indicating good model performance. With ArcGIS 10.4, the mean results of the MaxEnt replicates were used to generate a suitable habitat index (HSI) ranging from 0 to 1. Higher values indicate more suitable habitats. The HSI values were classified into four suitability levels: high suitability, moderate suitability, low suitability, and unsuitable. Geospatial analysis was performed in ArcGIS v10.8, and plotting was conducted in R v4.3.2.

### 2.4. Projection of Future Suitable Habitat

Future climate data were obtained from the BCC-CSM2.MR model, a high-resolution climate projection model developed under the Coupled Model Intercomparison Project Phase 6 (CMIP6). This model includes atmosphere, land surface, ocean and sea ice. The future scenario selected as the primary focus of this study was SSP245, an upgraded version of the medium-intensity SSP2 pathway, comparable to the RCP4.5 scenario. According to climate projections, RCP4.5 peaks around 2040 and stabilizes before 2080, which aligns with the future development trajectory of China. Accordingly, four future time periods were defined under SSP245: 2021–2040, 2041–2060, 2061–2080, and 2081–2100. To capture scenario uncertainty, additional projections were carried out under SSP126 (low emissions) and SSP585 (high emissions) for the 2081–2100 period.

For future projections, only the bioclimatic variables (Bio1–Bio19) from the BCC-CSM2-MR model under SSP245 were employed. NDVI, elevation, slope, and aspect were not included in these projections, as they are neither available nor reliably estimable for future scenarios; instead, they were restricted to the contemporary model and were not propagated forward. Consequently, the future suitability maps reflect changes driven solely by climate, ignoring future changes in vegetation and land use.

To assess extrapolation, we performed MESS analysis using the ‘mess’ function in the dismo R package. MESS values below zero indicate environmental conditions outside the training range, where predictions are less reliable. Further, to quantify uncertainty in projected area changes, suitable area was calculated for each of the 10 bootstrap replicates per species; the standard deviation (SD) across replicates was used as a measure of inter-model variability. Results are reported as mean ± SD in Section 3.

### 2.5. Centroid Shift in Suitable Habitats

To evaluate spatial changes in suitable habitats over time, we analyzed the shifts in the geographic centroids of suitable habitat for each dominant tick species from the present day to the time period of 2081–2100. First, the continuous suitable habitat index (HSI, 0–1) was converted into a binary presence/absence map using the threshold that maximizes training sensitivity plus specificity. This threshold is widely considered superior to arbitrary cutoffs such as 0.5 or the lowest presence threshold. The obtained thresholds were: *Hae. longicornis*: 0.42, *Hae. Concinna*: 0.37, *D. silvarum*: 0.48, and *I. persulcatus*: 0.35. Pixels with HSI ≥ threshold were classified as “suitable”.

Centroids (geographic centers) were calculated using the Mean Center tool in ArcGIS 10.4, weighting each suitable pixel by its HSI value. The direction and distance of centroid shifts between the current period and future periods were computed using the Point Distance analysis.

## 3. Results

### 3.1. Distribution of Tick Species in the Beijing–Tianjin–Hebei Region

A total of 1446 articles were retrieved, including 784 Chinese literature (462 from CNKI, 322 from Wanfang) and 662 foreign studies (633 from PubMed, 29 from Web of Science). After removing duplicates and articles with unclear or irrelevant distribution information (data cleaning), 40 articles (33 in Chinese and 7 in English) were screened for the final analysis (Figure S1).

**Figure 1:**
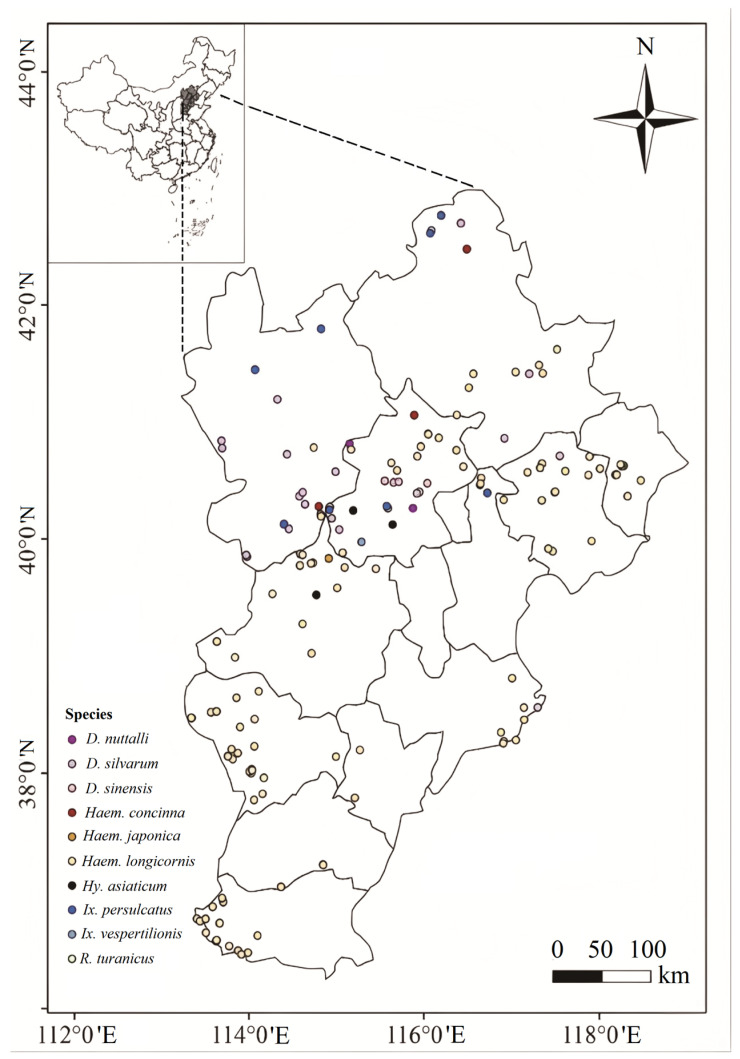
Geographic distribution of tick species in the BTH region. Different colors represent different tick species.

In addition, based on both our own field sampling records and occurrence data extracted from published literature, we compiled the distribution data of tick species in the BTH region. In total, 19 tick species belonging to 6 genera have been recorded to date across the BTH region, with distribution mapped at the prefecture level or below (Figure 1).

A total of 167 valid occurrence records were obtained from 55 publications. *Hae. longicornis* was found to be widely distributed in both plain and mountainous areas of the region, while other tick species were primarily recorded in the northern part of the BTH region. Based on the known distribution patterns of various tick species across different ecological habitats and the availability of occurrence data, we selected four dominant tick species in the region for subsequent ecological niche modeling analyses. The 167 records comprised: *Hae. longicornis* (116 records from 30 publications), *D. silvarum* (31 records from 12 publications), *I. persulcatus* (11 records from 10 publications), and *Hae. concinna* (9 records from 3 publications). All coordinates have been verified; the full dataset is available in Tables S1 and S2.

### 3.2. Environmental Variables Influence Tick Distribution

To construct species-specific MaxEnt models for different tick species, an initial set of 19 bioclimatic variables was considered. The species-specific subsets of variables were then selected based on correlation analysis. The contributions of environmental variables were assessed by using the Jackknife test, with the key influencing variables for each species summarized (Table S1).

Furthermore, to investigate the distribution characteristics of ticks, the environmental factors affecting the current and potential distribution of tick species had been analyzed (Figure S2A–D). The distribution of *Hae. longicornis* and *Hae. concinna* was mainly influenced by elevation, mean temperature of the coldest quarter (Bio11), slope, and NDVI. For *D. silvarum*, the key variables were elevation, slope, and NDVI. The distribution of *I. persulcatus* was primarily driven by Bio11 and aspect. The highest predicted suitability for *Hae. longicornis* occurred around 184.36 m in elevation and −4.50 °C in temperature, whereas for *D. silvarum* it occurred around 1180.58 m and −18.26 °C. Furthermore, the regularized training gain results of the MaxEnt models, as shown in the Jackknife plots (Figure S2A–D), revealed that when using single environmental variables, elevation contributed the highest gain for *Hae. longicornis*, followed by Bio11, slope, Bio3, and Bio16. For *Hae. concinna*, Bio11 exhibited the highest gain, followed by NDVI, slope, and Bio17. For *D. silvarum*, elevation had the highest gain, followed by Bio11, slope, Bio3, and Bio16. For *I. persulcatus*, Bio11 showed the highest gain, followed by aspect and Bio3. The Jackknife test further elucidated the relative contribution of each predictor (Figure S2A–D; Table S1). For *Hae. longicornis*, elevation provided the highest regularized training gain when used in isolation, followed by mean temperature of the coldest quarter (Bio11) and slope, suggesting that temperature and topographic heterogeneity are primary drivers of its broad distribution. In contrast, the distribution of *Hae. concinna* was most strongly governed by Bio11, with NDVI and slope also contributing substantially, indicating a narrower niche with higher moisture and vegetation requirements. For *D. silvarum*, elevation again exhibited the highest gain, together with slope and NDVI, reinforcing its affinity for montane habitats. In *I. persulcatus*, Bio11 was the dominant predictor, followed by aspect and Bio3 (isothermality), underscoring its sensitivity to cold-season temperatures and microclimatic conditions. These results, in line with the variable contribution analyses, confirm that temperature, elevation, and vegetation are the most influential factors defining the ecological niches of these four tick species within the BTH region. Based on the results of the Pearson correlation analysis, the final selected variables for modeling were as follows: for *Hae. longicornis*, ten variables were selected, including Bio2, Bio3, Bio11, Bio14, Bio15, Bio16, elevation, slope, aspect, and NDVI; for *Hae. concinna*, six variables were selected, including Bio3, Bio11, Bio17, slope, aspect, and NDVI; for *D. silvarum*, nine variables were selected, including Bio2, Bio3, Bio7, Bio12, Bio17, elevation, slope, aspect, and NDVI; and for *Ixodes persulcatus*, five variables were selected, including Bio3, Bio11, slope, aspect, and NDVI.

### 3.3. Suitable Habitat for Dominant Tick Species Under Current Climate Conditions

**Figure 2:**
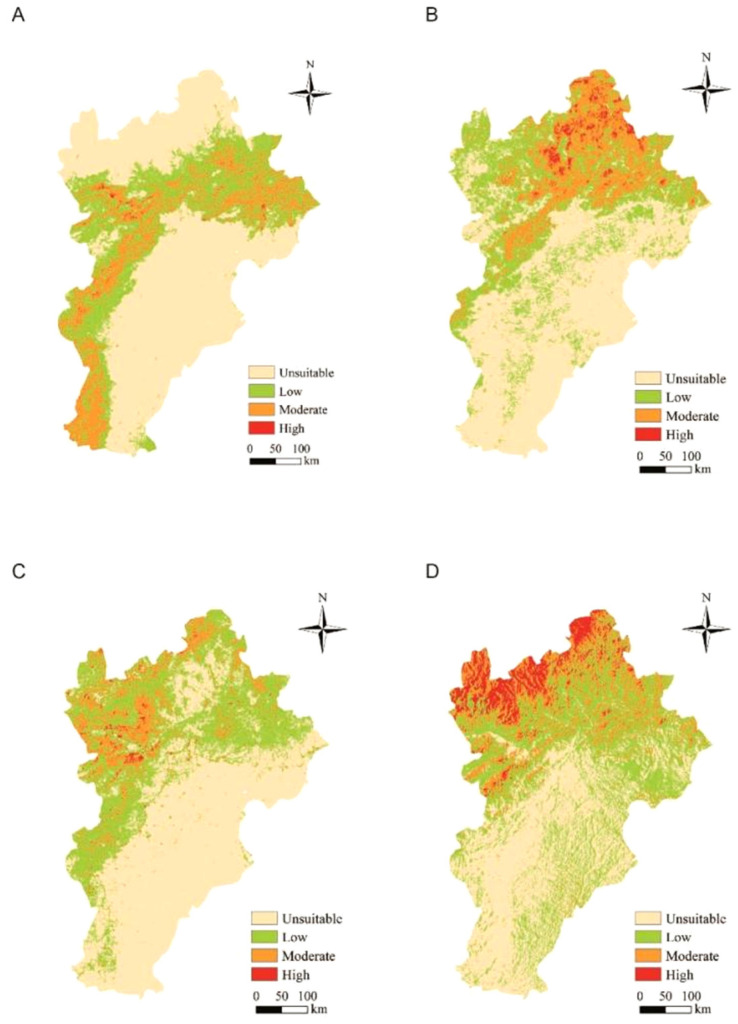
Predicted spatial distribution of tick suitable habitats under the current climate scenario in the BTH region. (**A**) *Hae. longicornis*; (**B**) *Hae. concinna*; (**C**) *D. silvarum*; (**D**) *I. persulcatus*.

Based on ten replicates of MaxEnt runs with 19 environmental variables, the MaxEnt models demonstrated good predictive performance for all four species, with test AUC values ranging from 0.86 to 0.91. The True Skill Statistic (TSS), Cohen’s kappa, and omission rates also indicated high model accuracy (TSS: 0.72–0.88) (Table S3). According to the AUC curve evaluation criteria, a higher AUC value for the test set indicates better model performance. In this study, the high AUC values obtained demonstrate that the predictive models performed well. Predicted suitable habitats for these species in the BTH region were classified into four categories: highly suitable, moderately suitable, low suitability, and unsuitable areas (Figure 2).

Under current climate, *Hae. longicornis* exhibited a broad belt-shaped distribution along the Taihang and Yanshan mountains, whereas *Hae. concinna*, *D. silvarum*, and *I. persulcatus* were largely confined to the northern mountainous areas (Zhangjiakou and Chengde). The southern plains were predicted as low or unsuitable for all four species (Figure 2).

### 3.4. Prediction of Spatial Distributions for Tick Species Under the Future Periods

**Figure 3:**
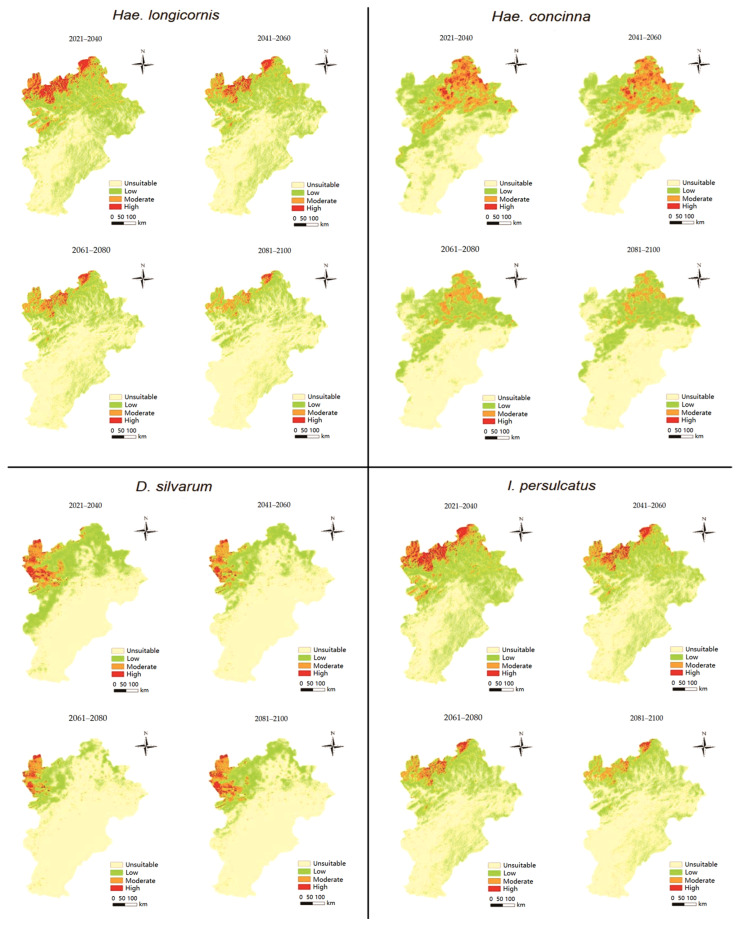
Projected changes in the spatial distribution of suitable habitats for *Hae. Longicornis*, *Hae. concinna*, *D. silvarum* and *I. persulcatus* in the BTH region from 2021 to 2100.

Under the SSP245 scenario, the four dominant tick species exhibited distinct but generally northward/northwestward shifts in their potential suitable habitats (Figure 3). *Hae. longicornis* was projected to expand its range steadily throughout the 21st century, with a net increase of approximately 35,583 km^2^ by 2081–2100, primarily in the Zhangjiakou and Chengde areas, while the southeastern plains would become largely unsuitable. In contrast, *Hae. concinna* showed a tendency toward habitat fragmentation, with high-suitability zones gradually shrinking and becoming more discontinuous in northern regions (Figure 3). *D. silvarum* remained largely confined to the northwestern montane areas (especially Zhangjiakou), with only a modest expansion of high-suitability habitat (433 km^2^ by 2081–2100), though inter-period fluctuations suggested potential climate-driven instability (Figure 3). *I. persulcatus* continued to be restricted to cooler northern and northwestern zones, with a projected total suitable area increase of 16,673 km^2^, driven primarily by expansion of moderately suitable areas (Figure 3). Collectively, these projections indicate persistent high-risk zones in the northern mountainous regions, underscoring the need for targeted, long-term surveillance of tick-borne diseases in the BTH region. To quantify inter-model variability, we further examined the coefficient of variation (CV = SD/mean) derived from 10 bootstrap replicates; the CV maps (Figure S3) indicated generally low uncertainty in core suitable areas, with higher variability in transitional zones and southeastern plains where suitability was marginal.

To assess the uncertainty of area change estimates, we calculated the total suitable area (mean ± SD) for each species across the 10 bootstrap replicates. The net change in suitable area by 2081–2100 was 35,583 ± 2451 km^2^ for *Hae. longicornis*, 433 ± 186 km^2^ for *D. silvarum*, and 16,673 ± 3024 km^2^ for *I. persulcatus*. However, due to the small sample sizes and the limitations of presence-only data, formal significance testing was not performed; the reported uncertainty ranges reflect inter-model variability from the bootstrap replicates.

To assess the influence of excluding NDVI and topographic variables from future projections, we conducted a sensitivity analysis comparing contemporary model predictions with and without these non-climatic predictors. The climate-only model yielded larger areas of suitable habitat across all four species (average overestimation: 8–15%), particularly in the southeastern plains and low-elevation valleys, where NDVI and elevation exert strong limiting effects in the full model. This discrepancy suggests that projections excluding these variables may overestimate range expansion, especially in areas where non-climatic factors currently constrain tick distributions. These results underscore that our future projections should be interpreted as climate-driven potential suitability rather than actual predicted distributions.

Furthermore, to assess the sensitivity of future projections to scenario uncertainty, we also performed projections under SSP126 (low emissions) and SSP585 (high emissions) for the 2081–2100 period (Table S4). Under SSP126, the centroid shifts were less pronounced, with *Hae. longicornis* shifting approximately 35 km northwest, *D. silvarum* approximately 42 km northwest, *I. persulcatus* approximately 30 km north, and *Hae. concinna* approximately 38 km northeast, and suitable area expansions were substantially smaller across all species. Under SSP585, the shifts were more pronounced, with *Hae. longicornis* shifting approximately 78 km northwest, *D. silvarum* approximately 95 km northwest, *I. persulcatus* approximately 68 km north, and *Hae. concinna* approximately 85 km northeast, with larger suitable area expansions and greater fragmentation of *Hae. concinna* habitats. Importantly, the directional trends remained consistent across all three scenarios, indicating that the general spatial pattern is robust to scenario uncertainty. However, the magnitude of range shifts varies substantially across scenarios, underscoring the importance of considering a range of future pathways in risk assessments.

### 3.5. Centroid Shifts in Suitable Habitats Across Future Periods

**Figure 4:**
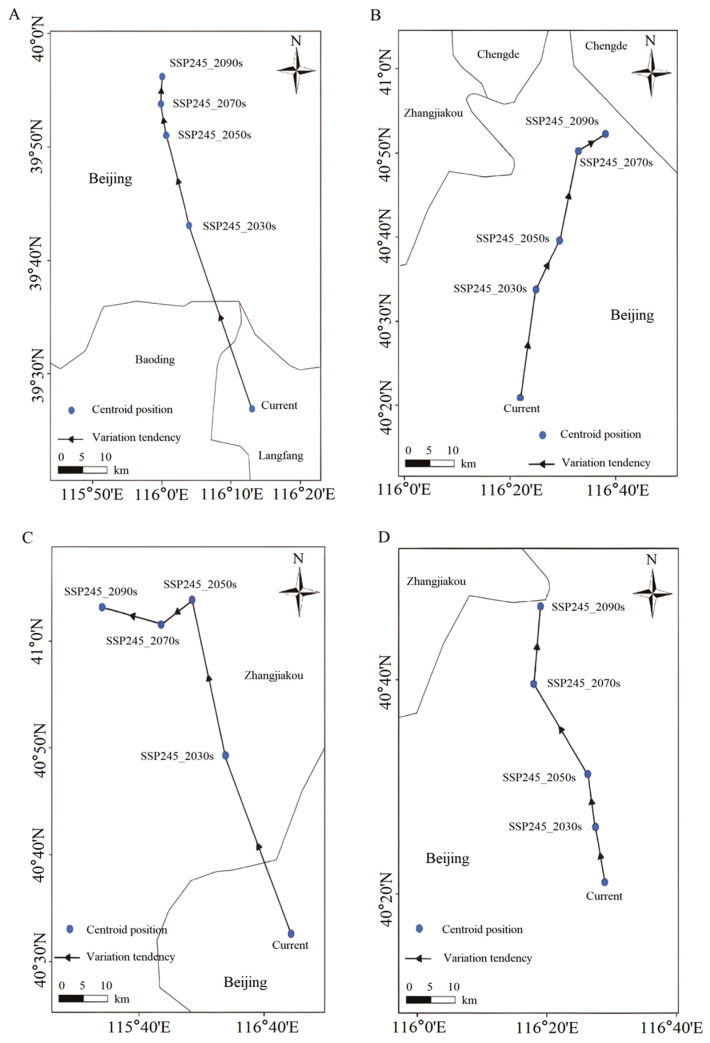
Changes in the centroid of the potential suitable habitats for the dominant tick species in BTH region. (**A**) 2021–2040; (**B**) 2041–2060; (**C**) 2061–2080; (**D**) 2081–2100.

To assess the future spatial dynamics of dominant tick species, the changes in the centroids of their potential suitable habitats across different time periods were further analyzed (Figure 4). By 2081–2100, the centroid of suitable habitat for *Hae. longicornis* was projected to shift approximately 57.6 km northwest, with a latitude increase of about 0.5° and a longitude change of 0.14°, indicating a trend of northward expansion. Similarly, *Hae. concinna* was expected to shift about 63.0 km to the northeast, with changes of approximately 0.4° in latitude and 0.5° in longitude, reflecting a northeastward expansion. The *D. silvarum* showed a projected centroid shift of around 71.1 km to the northwest, with a latitude change of 0.6° and a longitude change of 0.4°, suggesting a westward expansion of its suitable habitat. In contrast, *I. persulcatus* is expected to shift its centroid approximately 50.0 km northward, with a latitude change of 0.4° and only a minor change in longitude. However, its overall potential suitable habitat still demonstrated a westward expansion trend. These results indicated species-specific but consistent directional shifts under future climatic conditions.

### 3.6. Extrapolation Assessment for Future Projections

To evaluate the reliability of future suitability projections under the SSP245 scenario, we performed a multivariate environmental similarity surface (MESS) analysis comparing future climate conditions (2081–2100) against the training environment (current climate) for each species (Figure S4). The MESS maps revealed that the majority (>85%) of the BTH region maintained positive MESS values across all future periods, indicating that future climate conditions remained largely within the range of the training data, and thus predictions in these areas are considered reliable.

Negative MESS values, signaling novel environmental conditions with higher extrapolation uncertainty, were detected in localized patches, primarily in the southeastern plains (including parts of Cangzhou, Hengshui, and southern Baoding) and sporadically in the low-elevation valleys of the Yanshan foothills. These negative values were mainly associated with projected increases in winter temperatures (Bio11) and changes in precipitation seasonality (Bio15) exceeding the historical variability captured in the training dataset. The proportion of cells with negative MESS remained relatively stable across time periods (approximately 8–12% of total area), with a slight increase toward 2081–2100 (Figure S4).

The results reveal that the projected northward/northwestward centroid shifts and suitability expansions in the northern mountainous areas (Zhangjiakou and Chengde) are supported by environmental conditions analogous to the current training range. Conversely, predictions for the southeastern plains, already classified as low or unsuitable under current climate, should be interpreted with greater caution due to the higher extrapolation risk in these regions. The MESS results reinforce the overall credibility of our future projections for high-risk areas, while highlighting specific zones where novel climate conditions warrant further monitoring and model refinement.

## 4. Discussion

In this study, we systematically compiled tick occurrence records from the Beijing–Tianjin–Hebei (BTH) urban agglomeration and applied the MaxEnt model to predict current and future potential suitable habitats for four dominant species: *Hae. longicornis*, *Hae. concinna*, *D. silvarum*, and *I. persulcatus*. Our results revealed distinct distribution patterns, with *Hae. longicornis* being widely distributed across plains and mountains, whereas the other three species are largely confined to the northern and north-western mountainous areas (Zhangjiakou and Chengde). This spatial segregation is broadly consistent with previous national-scale surveys [2,3,27], and the predominance of *Hae. longicornis* further corroborates its broad ecological tolerance reported in other regions of China. Our findings are also in line with a recent study from Ningxia, which identified similar environmental determinants for dominant tick species.

The MaxEnt models exhibited robust predictive performance, confirming the suitability of this presence-only modeling approach for tick distribution inference [15,19,20]. Environmental driver analysis identified mean temperature of the coldest quarter (Bio11), elevation, and NDVI as the primary determinants shaping the distribution of these species. The response curves indicated clear niche differentiation among the four ticks. For instance, even congeneric *Hae. longicornis* and *Hae. concinna* showed markedly different elevation optima, highlighting that topographic gradients in the BTH region create distinct microclimatic refugia that filter species according to their thermal and moisture requirements. Temperature during the cold season emerged as a critical constraint, consistent with known physiological thresholds for tick overwintering survival and range limits [15]. NDVI, acting as a proxy for vegetation cover and surface moisture, exerted strong influence particularly on *Hae. concinna* and *D. silvarum*, underscoring the role of habitat structure and humidity in questing success and host-seeking behavior. Notably, elevation contributed the highest regularized gain for *Hae. longicornis* and *D. silvarum*. Rather than a direct driver, elevation serves as a proxy for covarying environmental gradients, including temperature lapse rates, moisture, forest structure, snow duration, and host communities, and its strong contribution reflects the combined influence of these factors on tick distribution.

The projected centroid shifts under the SSP245 scenario—northwestward for *Hae. longicornis*, *D. silvarum*, and *I. persulcatus*, and northeastward for *Hae. Concinna*, are broadly aligned with earlier modeling efforts that predicted poleward and upward range expansions of ticks in response to warming climates [17,28,29,30]. These directional shifts also resonate with recent field observations of *Haemaphysalis* and *Ixodes* species expanding into higher latitudes across East Asia, where milder winters have facilitated colonization of previously cooler zones. The congruence between our projections and these independent empirical records suggests that the observed trends likely reflect genuine climate-driven responses. Importantly, beyond climate, our projections should be interpreted as climate-driven potential suitability rather than forecasts of actual future tick distributions. Several non-climatic factors that were not included in our models may substantially influence future tick distributions. First, host availability is a critical determinant of tick population persistence, as ticks depend on vertebrate hosts for blood meals and reproduction. The availability and distribution of livestock, deer, rodents, and wild boar, all of which are known hosts for the four tick species examined here [2,30], may shift under climate change and land-use modification, creating mismatches between climatic suitability and actual host presence. Second, land-use change and urbanization are proceeding rapidly in the BTH region. Large-scale afforestation and ecological restoration, such as the Three-North Shelterbelt program which has substantially increased forest cover in northern Hebei over the past two decades, have created new wildlife habitats that may serve as corridors for tick dispersal [17]. Concurrently, rapid urban expansion fragments natural landscapes while creating novel peri-urban interfaces where domestic animals, wildlife, and humans intersect, potentially increasing the risk of tick-borne pathogen spillover [31]. Third, dispersal limitations were not considered; our projections assume unlimited dispersal, which may overestimate range expansion for species with limited mobility or fragmented habitat connectivity. Fourth, future vegetation changes are not captured in our climate-only projections, despite evidence that vegetation structure strongly influences tick questing success and survival in the contemporary model. Climate-only projections are known to overestimate range expansions because they ignore the lagged responses of vegetation to climate change and the constraints imposed by land-use change [32]. Fifth, dispersal barriers such as major roads, agricultural fields, and urban areas may further limit tick spread beyond climate-based projections. Collectively, these omissions highlight that our future projections should be interpreted as plausible directions of climate-driven change rather than precise predictions of future distributions. Moreover, the projected fragmentation of *Hae. concinna* suitable habitats deserves particular attention. This pattern aligns with the hypothesis that warming can induce vegetation-climate decoupling, whereby rising temperatures modify vegetation structure and create suboptimal microhabitats for this moisture-sensitive species. Concurrent urban expansion and infrastructure development could further fragment continuous habitats, as also observed in projections of climate-driven suitability changes for other taxa in northern China, although these factors were not directly modeled in our study [32]. These findings highlighted the need to consider both climatic and non-climatic drivers in future risk assessments.

Given the emphasis on One Health, we argue that a comprehensive risk assessment must address not only climatic suitability but also the interplay among tick microbiome composition, host community dynamics, and pathogen circulation [10,33,34]. While our suitability maps identify climatically favorable areas for ticks, actual transmission risk hinges on three additional factors operating at the ecology-microbiology-public health interface. First, the tick microbiome can enhance or suppress pathogen transmission through immune modulation or resource competition, with effects that vary by temperature and humidity. Second, host availability and competence, shaped by climate and land-use change, determine whether ticks complete their life cycles and maintain transmission cycles. Third, pathogen presence relies on multi-host networks that are not captured by abiotic models. Consequently, a climate-suitable area may pose low risk if competent microbiomes or reservoir hosts are absent, whereas a moderately suitable area could become high-risk as an interface between wildlife, domestic animals, and humans. In the BTH region, ongoing ecological restoration and urban expansion are creating new wildlife habitats and human–wildlife interfaces that our climate-driven models do not capture. Future work integrating microbiome profiling, host surveys, and pathogen surveillance with our suitability maps would enable more comprehensive risk assessment and targeted One Health interventions.

Several limitations must be acknowledged when interpreting our results. First, occurrence data were primarily derived from published literature and GBIF, which are subject to inherent sampling biases. Although we applied spatial rarefaction to mitigate this, residual bias cannot be entirely excluded, and under-representation of certain habitats or seasons remains possible. Second, the MaxEnt model inherently accounts only for abiotic environmental factors; biotic interactions, host distribution and abundance, and human activities were not included, which may explain some presences in areas predicted as marginally unsuitable. The absence of explicit host distribution modeling is particularly relevant, as tick persistence depends on the availability of suitable vertebrate hosts, including livestock, deer, rodents, and wild boar, whose populations and ranges may also shift under climate change and land-use modification. Third, the choice of the binary threshold for defining “suitable” habitat, while methodologically standard, is one of several acceptable alternatives; using different thresholds would alter the absolute extent of suitable areas and centroid positions, although the overall directional trends are likely robust. Fourth, although we provide multiple performance metrics, the AUC values reported here are strictly valid for within-species model comparison and should not be over-interpreted across species; the complementary TSS, kappa, and omission rates support the reliability of our models. In addition, the small sample sizes for *Hae. concinna* (*n* = 9) and I. persulcatus (*n* = 11) warrant particular caution; while MaxEnt can produce informative models with as few as 10–15 records when carefully validated, predictions for these two species should be interpreted with greater caution. Fifth, the decadal projections lack long-term field validation data, and therefore remain to be verified by future longitudinal monitoring. Sixth, and crucially, our future projections were driven solely by climatic variables (Bio1–Bio19), because NDVI, elevation, slope, and aspect cannot be reliably projected for future time periods. Consequently, our future suitability maps reflect only climate-driven changes and may oversimplify the actual response of tick habitats, particularly given that vegetation dynamics and land-use shifts are expected to co-evolve with climate [35]. The sensitivity analysis revealed that climate-only models overestimate suitable areas by 8–15% compared with full models including NDVI and topographic variables, consistent with previous findings showing that climate-only projections tend to overestimate range expansions because they do not account for the lagged responses of vegetation, constraints imposed by land use, or the availability of suitable host populations [36]. Moreover, we did not incorporate land-use change projections, despite ongoing urban expansion and ecological restoration in the BTH region, both of which could substantially modify local suitable habitat and dispersal corridors. Additionally, the future climate projections rely on a single model (BCC-CSM2-MR) as the primary scenario; this single-model approach does not capture the full range of climate model uncertainty, and ensembles of multiple models would provide a more robust assessment of future variability. Recent ecological modelling guidelines emphasize that transparent communication of such uncertainties, including model structure, scenario, and data limitations, is essential for the credible application of species distribution models in policy and public health contexts. Collectively, these caveats underscore that our projections should be interpreted as indicative trends rather than precise forecasts, and they highlight the need for integrated, multi-factor modelling frameworks in future work, particularly those incorporating multi-model ensembles, dynamic vegetation and land-use scenarios, as well as explicit host distribution data.

Despite these limitations, this study provides the first systematic spatial assessment of dominant tick species in the BTH region based on an integrated dataset of literature and field records. The identification of persistent high-risk areas and the predicted northward/northwestward centroid shifts offer actionable, spatially explicit targets for public health agencies. In particular, targeted tick surveillance should be prioritized in the expansion zones of *Hae. longicornis*, given its vector competence and the confirmed presence of associated pathogens in the region [37,38]. As climate change continues to reshape the landscape of tick-borne disease risk, our findings establish a reproducible, data-driven baseline that can guide evidence-based resource allocation and early-warning system development in the BTH region.

## 5. Conclusions

In summary, this study compiled 167 occurrence records of ticks from the Beijing–Tianjin–Hebei (BTH) region and identified 19 tick species, with *H. longicornis*, *H. concinna*, *D. silvarum*, and *I. persulcatus* being the dominant species. The MaxEnt model (AUC: 0.86–0.91; TSS: 0.72–0.88), temperature (Bio11), elevation, and NDVI were found to be the primary environmental drivers of their distributions. Under the SSP245 climate scenario (2081–2100), the suitable habitat centroids are projected to shift northwestward for *Hae. longicornis*, *D. silvarum*, and *I. persulcatus*, and northeastward for *Hae. concinna*. These results provide a spatially explicit baseline for tick surveillance in the BTH region. However, it is essential to emphasize that the future projections considered only climate variables and should be interpreted as climate-driven potential suitability rather than forecasts of actual future distributions. The absence of a future NDVI, land-use change, host availability, urbanization, and dispersal dynamics from our models means that the projected range shifts represent plausible directions of climate-driven change, but the absolute magnitudes and specific spatial patterns may be modified by these unmodeled factors. Therefore, these projections are most appropriately used for identifying priority surveillance areas and guiding resource allocation, rather than as precise predictions of future tick presence. Integrated modeling frameworks incorporating dynamic vegetation, land-use scenarios, and host distribution data are needed to refine these projections. Despite these limitations, this study offered a reproducible and data-driven foundation for prioritizing high-risk areas and guiding regional tick-borne disease prevention strategies.

## Data Availability

The datasets used and analyzed during the current study are available from the corresponding author on reasonable request.

## Supplementary Materials

The following supporting information can be downloaded at: https://www.mdpi.com/article/10.3390/vetsci13070651/s1, Figure S1. Study design and data sources. Data were collected from a field survey, reference book and literature review. Figure S2. Construction of the MaxEnt model. (A–H) The receiver operating characteristic curve (ROC) predicting the current potential distribution of different tick species based on MaxEnt model, corresponding to *Hae. longicornis*, *Hae. concinna*, *D. silvarum* and *I. persulcatus*, respectively. Figure S3. Coefficient of variation (CV) maps of MaxEnt predictions for four dominant tick species (*Haemaphysalis longicornis*, *Haemaphysalis concinna*, *Dermacentor silvarum*, and *Ixodes persulcatus*) based on 10 bootstrap replicates. Figure S4. Multivariate environmental similarity surface (MESS) analysis for four dominant tick species (*Haemaphysalis longicornis*, *Haemaphysalis concinna*, *Dermacentor silvarum*, and *Ixodes persulcatus*) comparing future climate conditions (2081–2100, SSP245) with the current training environment in the Beijing–Tianjin–Hebei region. Table S1. Percent contribution and permutation importance of key environmental variables for each tick species. Table S2. Occurrence records of tick species in the Beijing–Tianjin–Hebei region compiled from published literature and field collections. Table S3. Model performance metrics (mean ± SD) for each tick species. Table S4. Projected centroid shifts and suitable area changes under different SSP scenarios (2081–2100).
