# Supplementary material for: Diversity and Spatiotemporal Atlas of Ticks in the Beijing–Tianjin–Hebei Urban Agglomeration Based on the MaxEnt Model
Source: Vet Sci. 2026 Jul 3;13(7):651. doi: 10.3390/vetsci13070651 (PMC13431307; doi:10.3390/vetsci13070651)
Supplement: Supplementary file 1 [file vetsci-13-00651-s001.zip › Supplementary Figure S3.pdf]

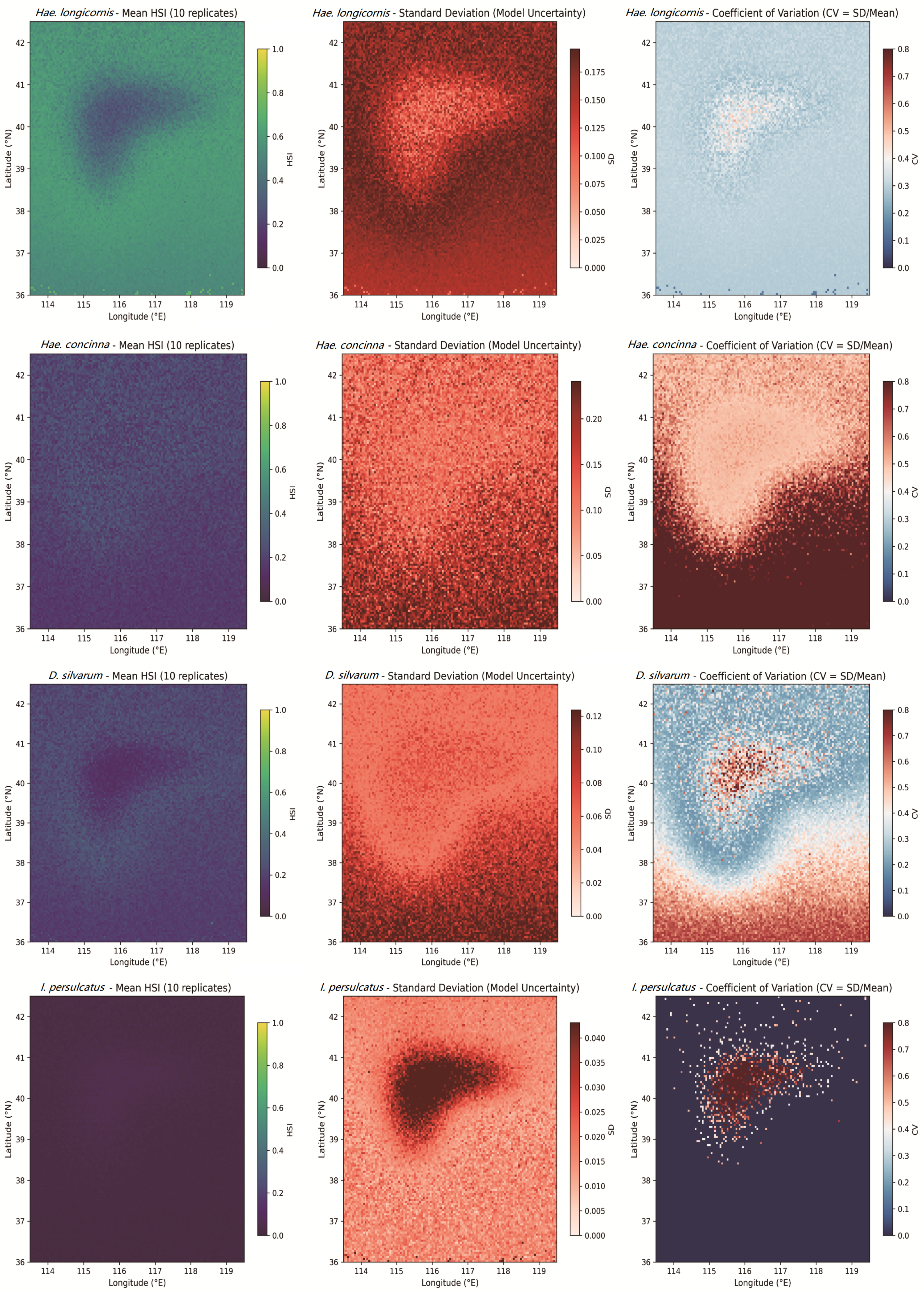
Hae. longicornis - Mean HSI (10 replicates)
Hae. longicornis - Standard Deviation (Model Uncertainty)
Hae. longicornis - Coefficient of Variation (CV = SD/Mean)
Hae. concinna - Mean HSI (10 replicates)
Hae. concinna - Standard Deviation (Model Uncertainty)
Hae. concinna - Coefficient of Variation (CV = SD/Mean)
D. silvarum - Mean HSI (10 replicates)
D. silvarum - Standard Deviation (Model Uncertainty)
D. silvarum - Coefficient of Variation (CV = SD/Mean)
I. persulcatus - Mean HSI (10 replicates)
I. persulcatus - Standard Deviation (Model Uncertainty)
I. persulcatus - Coefficient of Variation (CV = SD/Mean)
Latitude (°N)
Longitude (°E)
HSI
SD
CV
