# Supplementary figures and images for: Diversity and Spatiotemporal Atlas of Ticks in the Beijing–Tianjin–Hebei Urban Agglomeration Based on the MaxEnt Model
Source: Vet Sci. 2026 Jul 3;13(7):651. doi: 10.3390/vetsci13070651 (PMC13431307; doi:10.3390/vetsci13070651)

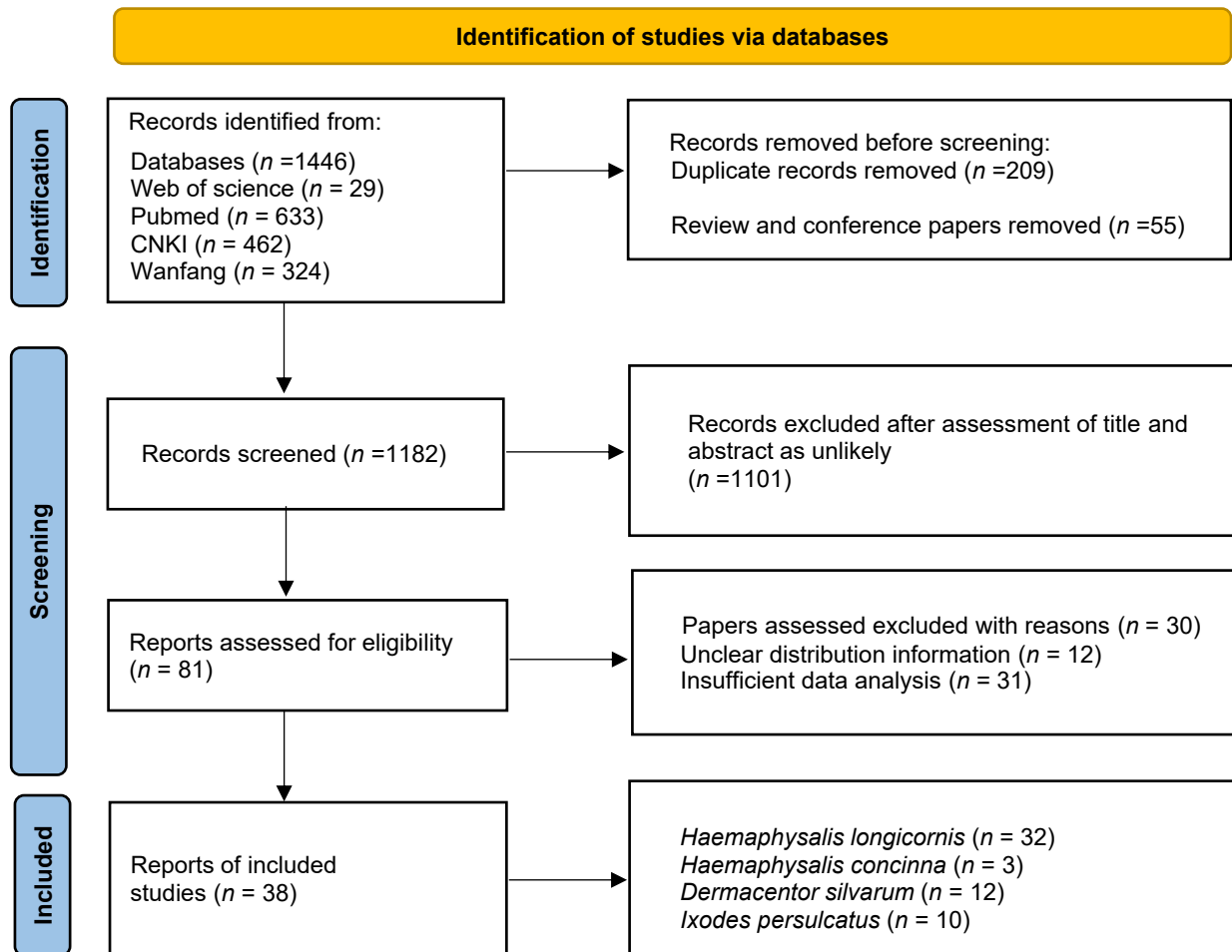

Supplement: Supplementary file 1 [file vetsci-13-00651-s001.zip › Supplementary Figure S1.pdf]

A

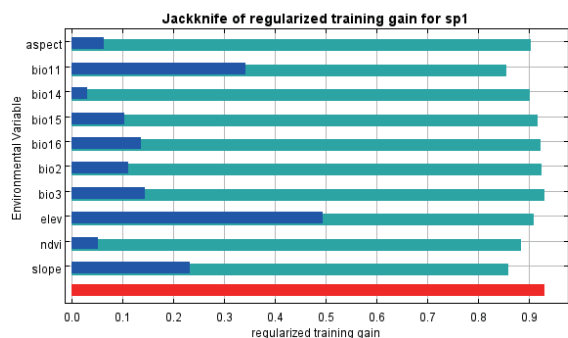

B

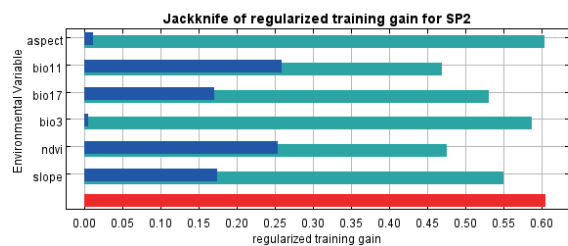

C

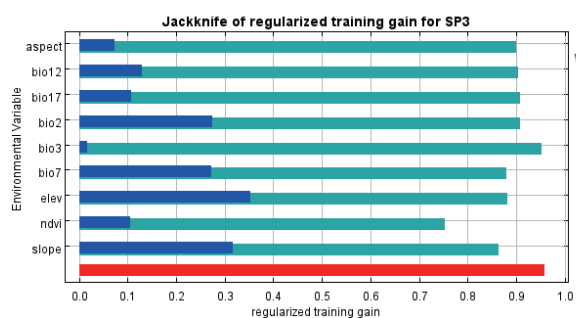

D

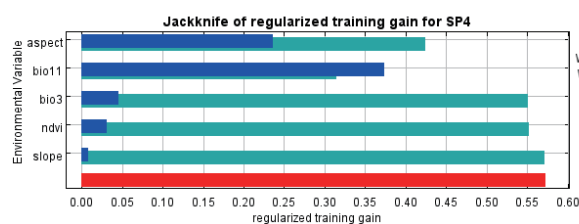

E

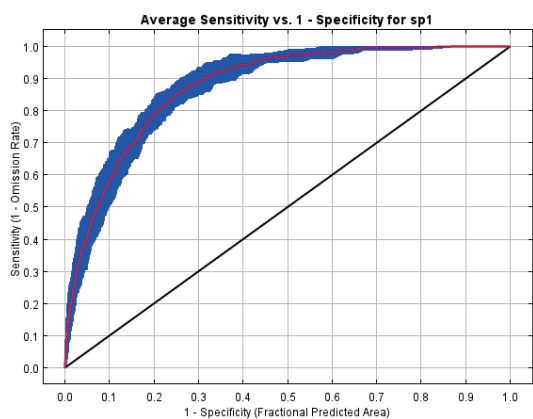

F

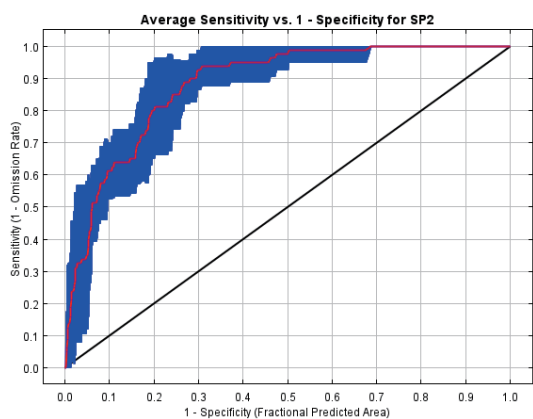

G

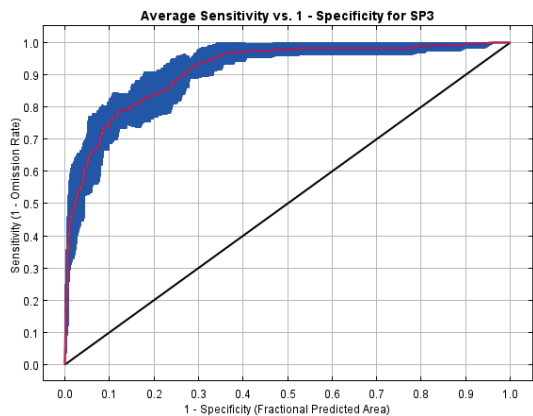

H

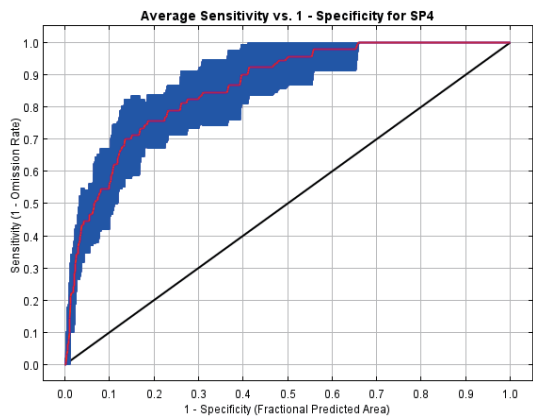

Supplement: Supplementary file 1 [file vetsci-13-00651-s001.zip › Supplementary Figure S2.pdf]

***Hae. longicornis***

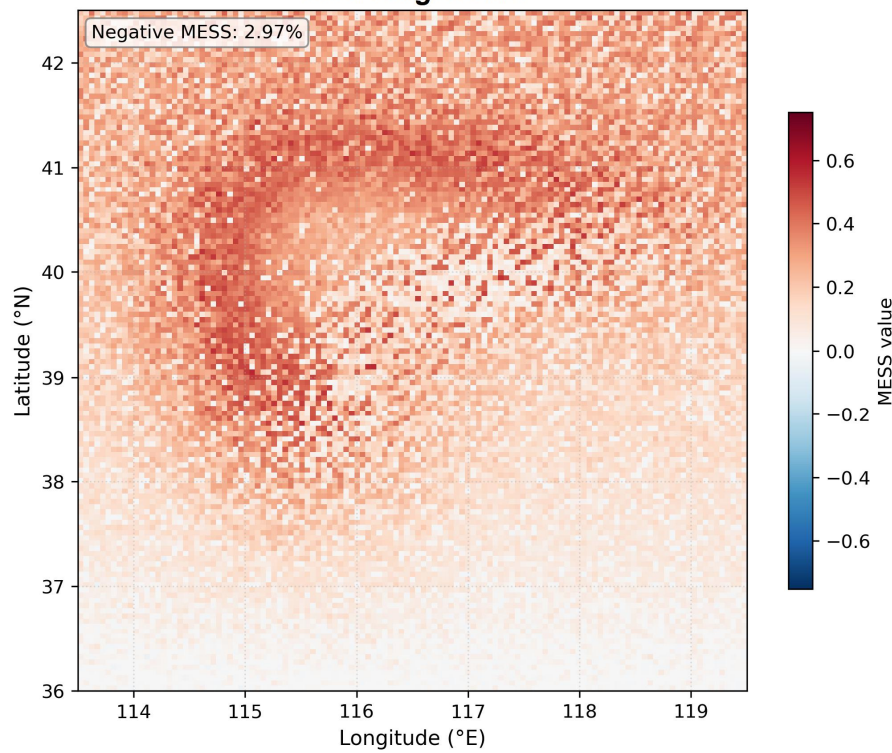

***Hae. concinna***

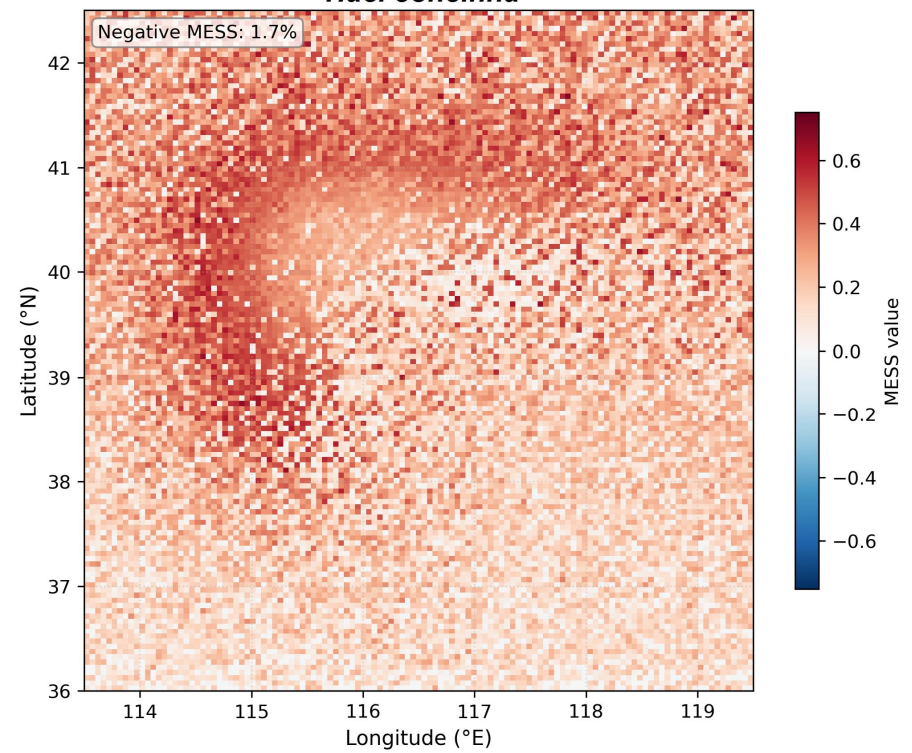

***D. silvarum***

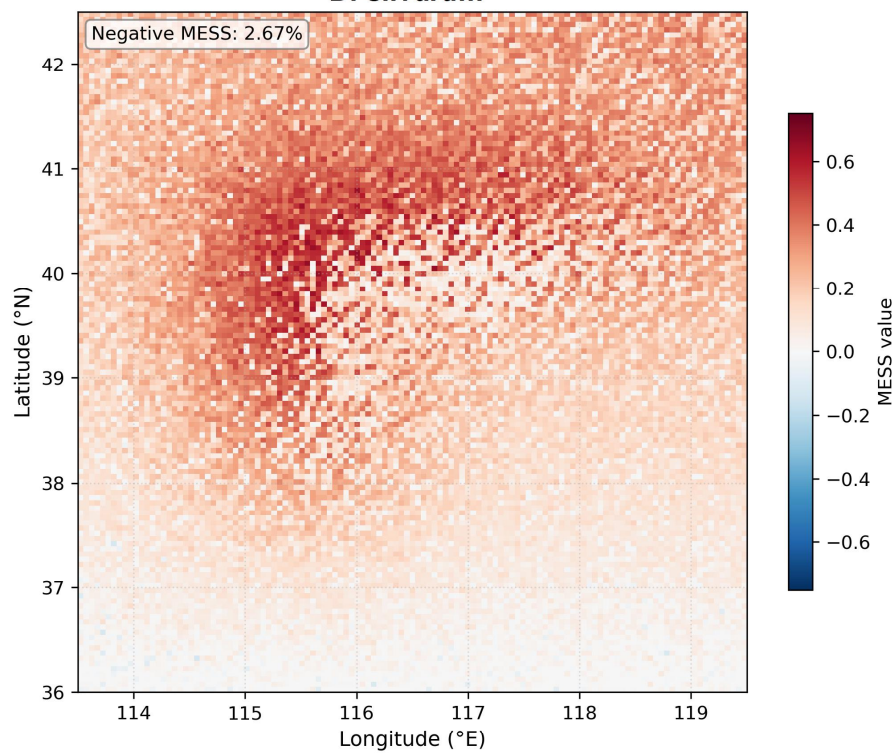

***I. persulcatus***

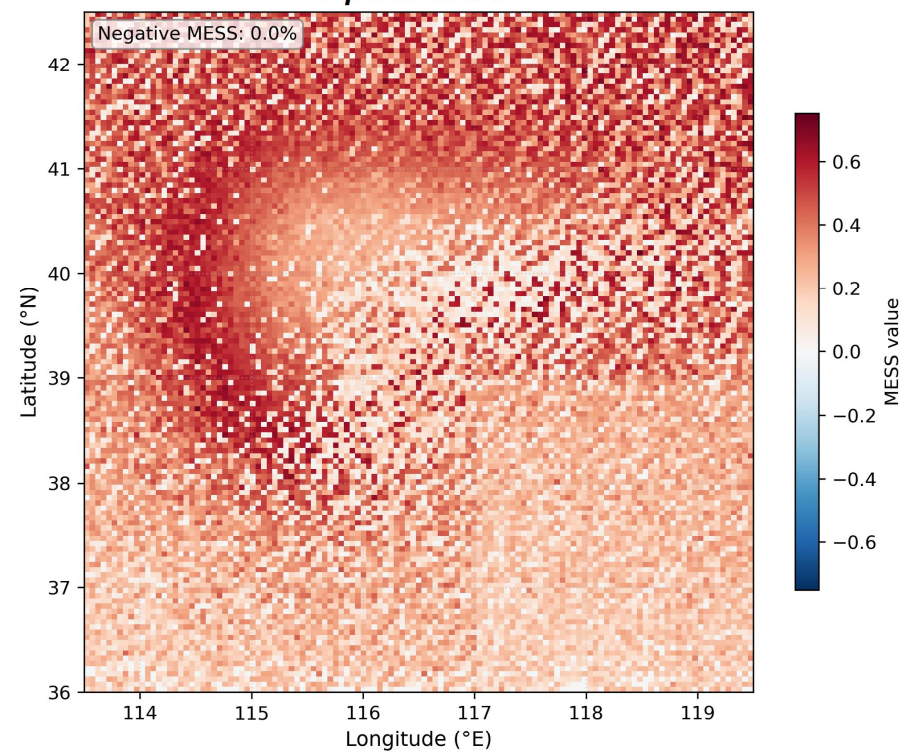

Supplement: Supplementary file 1 [file vetsci-13-00651-s001.zip › Supplementary Figure S4.pdf]
